# Prevalence and characteristics of S-point bleeding compared to non S-point bleeding in severe epistaxis

**DOI:** 10.1016/j.bjorl.2020.07.008

**Published:** 2020-09-12

**Authors:** Hamin Jeong, BoYoon Choi, Jiyeon Lee, Kyung Soo Kim, Sung Jin Min, Jin Kook Kim

**Affiliations:** a Konkuk University Medical Center, Konkuk University School of Medicine, Department of Otorhinolaryngology-Head and Neck Surgery, Seoul, Republic of Korea; b Chung-Ang University, College of Medicine, Department of Otorhinolaryngology-Head and Neck Surgery, Seoul, Republic of Korea

**Keywords:** Epistaxis, Nasal septum, Emergency, Body mass index, Anemia

## Abstract

**Introduction:**

Stamm's S-point is gaining importance as a bleeding focus in severe epistaxis. However, prevalence and features of S-point bleeding compared to non S-point bleeding have not been studied.

**Objective:**

To investigate the characteristics of patients with S-point bleeding among those with severe epistaxis and to compare the factors involved in the treatment of epistaxis.

**Methods:**

We retrospectively analyzed medical records of 268 patients admitted to the otorhinolaryngology department of Konkuk University Hospital and Chung-Ang University Hospital with epistaxis of which the bleeding focus clarified. Patients with anterior nasal bleeding (n = 129) were excluded. The study was conducted at the department of otorhinolaryngology from January 2008 to August 2019. Collected data included patients’ demographic information, bleeding focus, body mass index underlying medical and sinonasal diseases, laboratory test results (initial hemoglobin, platelet count, and triglyceride level), use of anticoagulants, direction of epistaxis, initial and final treatments, and need for blood transfusion.

**Results:**

The prevalence of S-point bleeding was 28.8% of non-anterior bleeding cases. Mean body mass index score was lower in the S-point group (23.41 ± 3.71) compared to the non S-point group (24.93 ± 3.97) (*p* = 0.039). Underweight patients tended to show a greater incidence of S-point bleeding (15.0%) than non S-point bleeding (2.0%) (*p* = 0.010). Incidence of anemia was higher in the S-point group (67.5%) than in the non S-point group (36.4%). Anemia (Odds ratio [OR]: 3.635; 95% confidence interval [CI]: 1.669-7.914, *p* = 0.001) and underweight (body mass index < 18.5, OR: 8.559, CI: 1.648-44.445, *p* = 0.011) were significantly associated with S-point bleeding.

**Conclusion:**

Prevalence of S-point bleeding was significant, underlining the importance of examining the S-point in patients with severe epistaxis. Patients with S-point bleeding had lower body mass index scores and a higher incidence of anemia than those with non S-point bleeding.

## Introduction

Epistaxis is a common complaint and one of the most common reasons for visiting the emergency center in the otorhinolaryngology department; 60% of these patients suffer from epistaxis and 6% of them need medical service.^1^ Severe epistaxis is less common. However, since severe epistaxis is potentially life-threatening, urgent treatment in the otolaryngology department including hospitalization is required in up to 4% of the case.^2^

Definition of severe epistaxis is controversial. However, epistaxis that requires surgery, intervention, or blood transfusion can be defined as severe epistaxis. Severe epistaxis is known to originate from posterior bleeding, but the original bleeding focus was not identified in up to 50% of the severe epistaxis or recurrent epistaxis cases.^3^, ^4^ The usual surgical treatment for severe epistaxis involves cauterization of the branches of the Sphenopalatine Artery (SPA) or ligation of SPA and has a high rate of success. However, sometimes an approach involving the Anterior Ethmoidal Artery (AEA) is required.^3^

**Figure 1:**
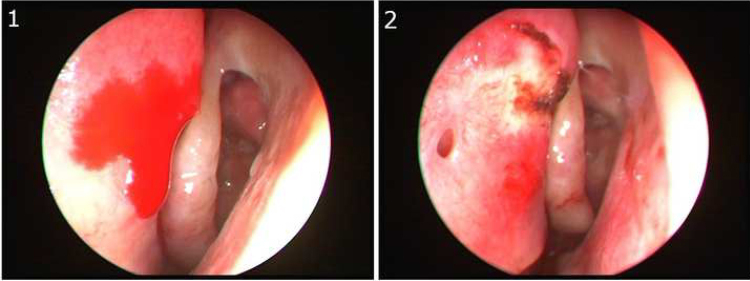
(1) Endoscopic view of left nasal cavity. Bleeding at Superior portion of nasal septum, above the axilla of middle turbinate (S-point). (2) S-point cauterization was performed.

Although SPA is a more common bleeding focus in severe epistaxis than AEA, the superior portion of the septum, which is supplied by AEA, is an important site of severe epistaxis. Bleeding from AEA can be spontaneous bleeding, post-sinus surgery bleeding, or traumatic bleeding. Recently, Kosugi et al. defined the Stamm's S-point as a bleeding point from the arterial vascular pedicle in the upper nasal septum, around the axilla of the middle turbinate, posterior to the septal body (Fig. 1).^2^

Endoscopic intervention has several benefits in controlling severe epistaxis in many patients. However, precise endoscopic examination is not easy during active bleeding. The upper nasal septum and consequently the S-point may be difficult to access endoscopically due to its much superior location in the nasal cavity. During active bleeding, blood flows posteriorly and laterally, which may be misdiagnosed as posterior bleeding.^2^ However, the prevalence of S-point bleeding that can be diagnosed incorrectly as posterior bleeding and features compared to non S-point bleeding have not been studied.

The purpose of this study was to investigate the prevalence and characteristics of patients with S-point bleeding among patients admitted with severe epistaxis and to compare the factors involved in the treatment of epistaxis.

## Methods

### Ethical committee approval

This study was approved by Konkuk University Hospital Institutional Review Board (Approval n° 2019-08-012-003) and Chung-Ang Universtiy Hospital Institutional Review Board (Approval n° 1912-023-19296).

### Subjects

Retrospective chart review was performed in 268 patients admitted to the otorhinolaryngology departments of Konkuk University Hospital and Chung-Ang University Hospital (both tertiary care hospitals) with a diagnosis of epistaxis of which the bleeding focus was clarified. The number of patients whose bleeding focus had not been clearly identified was 30. Therefore, these 30 patients were not included from the data collection. Data were collected from January 2008 to August 2019. Patients diagnosed with anterior nasal bleeding (i.e., from Kisselbach's plexus and Little's area) were excluded. The study group (the S-point bleeding group) included patients with the superior nasal septum as the bleeding focus (suspected S-point bleeding). The control group (the non S-point bleeding group) included patients in whom the superior nasal septum was not identified as the bleeding focus. Collected data included patients’ demographic information, bleeding focus, Body Mass Index (BMI), underlying medical and sinonasal diseases, laboratory test results (initial hemoglobin, platelet count, and triglyceride level), use of anticoagulants, direction of epistaxis, initial and final treatments, and need for blood transfusion.

### Statistical analysis

Independent *t*-test was used for comparisons of the interval scale data. Chi-squared test, Fisher's exact test, and regression analysis were performed for comparisons of the categorical data. Statistical significance was set at a *p*-value of 0.05. IBM SPSS Statistics ver. 25.0 program (IBM Co., Armonk, NY, USA) was used for significance testing. For all statistical tests, a *p*-value < 0.05 was considered to be statistically significant.

## Results

### Prevalence & demographic distribution

**Figure 2:**
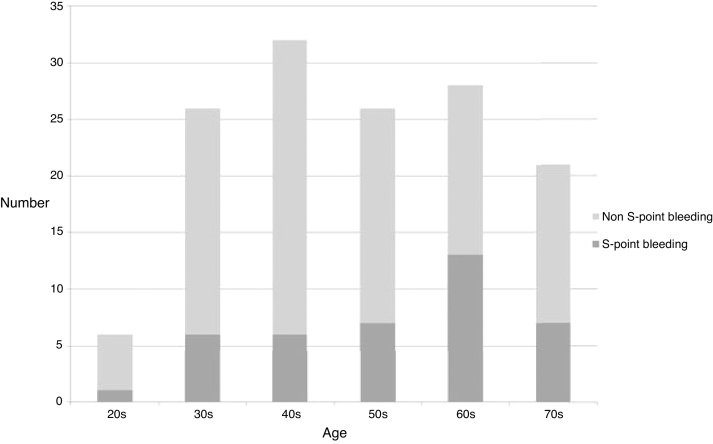
Age distribution of S-point bleeding group and non S-point bleeding group.

During the 11-year study period, 268 patients with epistaxis were admitted to two medical centers due to epistaxis. Of these, 139 patients (51.9%) with non-anterior bleeding were included in the study. 40 patients (28.8%) were included in the S-point group and 99 patients (71.2%) were included in the non S-point group. In our study, bleeding points of 51.9% of patients who were admitted due to epistaxis was non-anterior site. The prevalence of S-point bleeding in case of non-anterior bleeding was 28.8%. The age distribution of patients in this study is shown in Fig. 2. The age distribution was not deviated and the difference in age distribution was not statistically significant (*p* = 0.254). Both the groups demonstrated a male predominance. The mean age (mean ± standard deviation) was 55.90 ± 13.8 in the S-point group and 51.54 ± 16.0 in the non S-point group. However, the difference was not statistically significant (*p* = 0.132) (Table 1).

**Table 1:** Demographic distribution for S-point group and Non S-point group.

	**S-point bleeding (n** **=** **40, 28.8%)**	**Non S-point bleeding (n** **=** **99, 71.2%)**	**Total (n** **=** **139)**
**Mean age (±SD)**	55.90 (±13.8)	51.54 (±16.0)	52.79 (±15.4)
**Sex**			
Male	29	64	93
Female	11	35	46

### Underlying diseases & other characteristics

Comparison of the patient characteristics of the S-point group and the non-S-point group is shown in Table 2. Hypertension was noted in 32.5% (n = 13) of the patients in the S-point group and in 38.4% (n = 38) of the patients in the non S-point group. Diabetes *mellitus* was noted in 12.5% (n = 5) patients in the S-point group and in 6.1% (n = 6) of the patients in the non S-point group. 10% (n = 4) of the patients in the S-point group and 10.1% (n = 10) of the patients in the non S-point group were anticoagulant users. Bleeding was noted on the left side in 55% (n = 22) and 58.6% (n = 58) of the patients in the S-point group and the non S-point group, respectively. Sinonasal diseases such as septal deviation and rhinosinusitis were found in 50% (n = 20) of the patients in the S-point group and in 40.4% (n = 40) of the patients in the non S-point group. Dyslipidemia was noted in 7.5% (n = 3) of the patients in the S-point group and in 11.1% (n = 11) of the patients in the non S-point group. Thrombocytopenia was noted in 2.5% (n = 1) and 3% (n = 3) of the patients in the S-point group and the non S-point group, respectively. However, none of these characteristics showed a statistically significant difference.

**Table 2:** Characteristics and treatments of S-point bleeding and Non S-point bleeding.

**Characteristics**	**S-point bleeding (n** **=** **40)**	**Non S-point bleeding (n** **=** **99)**	***p*****-value**
HTN	13 (32.5%)	38 (38.4%)	0.515
DM	5 (12.5%)	6 (6.1%)	0.203
Anticoagulants	4 (10.0%)	10 (10.1%)	1
Direction			0.699
Left	22 (55.0%)	58 (58.6%)	
Right	18 (45.0%)	41 (41.4%)	
Sinonasal disease	20 (50.0%)	40 (40.4%)	0.301
Dyslipidemia	3 (7.5%)	11 (11.1%)	0.757
Thrombocytopenia	1 (2.5%)	3 (3.0%)	1
Initial treatment			0.550
Nasal packing	20 (50.0%)	57 (57.6%)	
Cauterization (L/A)	10 (25.0%)	25 (25.3%)	
Observation	10 (25.0%)	17 (17.2%)	
Final treatment			0.763
Nasal packing	3 (7.5%)	12 (12.1%)	
Cauterization (L/A)	10 (25.0%)	17 (17.2%)	
Observation	7 (17.5%)	18 (18.2%)	
Cauterization (G/A)	17 (42.5%)	40 (40.4%)	
Embolization	3 (7.5%)	12 (12.1%)	

L/A, Local Anesthesia; G/A, General Anestesia.

Univariate regression analysis was performed to identify the risk of S-point bleeding. The results are presented in Table 3. Anemia (Odds ratio [OR = 3.635]; 95% Confidence Interval [95% CI: 1.669–7.914], *p* = 0.001) and underweight (BMI < 18.5, OR = 8.559, 95% CI: 1.648–44.445, *p* = 0.011) showed a significant association with S-point bleeding. Other characteristics did not show a statistically significant association.

**Table 3:** Univariable logistic regression analysis of S-point bleeding.

	**OR (95% CI)**	***p*****-value**
**Anemia**	3.635 (1.669–7.9140)	0.001^a^
**Underweight**	8.559 (1.648–44.445)	0.011^a^
**HTN**	0.773 (0.356–1.679)	0.515
**DM**	2.214 (0.635–7.720)	0.212
**Anticoagulants**	0.989 (0.291–3.358)	0.986
**Direction**	1.157 (0.552–2.426)	0.699
**Sinonasal disease**	1.475 (0.705–3.087)	0.302
**Dyslipidemia**	0.649 (0.171–2.460)	0.525
**Thrombocytopenia**	0.821 (0.083–8.131)	0.866

OR, Odds Ratio; 95% CI, Confidence Interval.

Underweight, BMI < 18.5.

a Indicate statistically significant (*p*-value < 0.05).

### Initial and final treatments for S-point bleeding and non-S-point bleeding

Nasal packing was the initial treatment in 50% (n = 20) of the patients with S-point bleeding and in 57.6% (n = 57) of the patients with non S-point bleeding. Bipolar electric cauterization under local anesthesia was performed in 25.0% (n = 10) and 25.3% (n = 25) of the patients in the S-point group and the non S-point group, respectively. Observation was the initial treatment in 25% (n = 10) and 17.2% (n = 17) of the patients in the S-point group and the non S-point group, respectively. The difference in initial treatment was not statistically significant between the groups (*p* = 0.550) (Table 2).

Final treatments for the two groups are shown in Table 2. Cauterization under local anesthesia was performed in 25.0% (n = 10) and 17.2% (n = 17) of the patients in the S-point group and non S-point group. 42.5% (n = 17) and 40.4% (n = 40) patients in the S-point group and non S-point group underwent cauterization under general anesthesia for final treatment. Cauterization was the most common final treatment in both the groups, and the difference between the two groups was not statistically significant (*p* = 0.763).

In case of 30 patients whose bleeding focus was not identified, if active bleeding did not exist, observation was performed. In the presence of active bleeding, nasal packing was performed.

### Relationship between S-point bleeding and BMI

Difference in the BMI distribution for the two groups is presented in Table 4. Underweight (BMI < 18.5) was noted in 15.0% (n = 6) of the patients in the S-point group and in 2.0% (n = 2) of the patients in the non S-point group. BMI was within the normal range (18.5 to 23) in 20.0% (n = 8) of the patients in the S-point group and in 28.3% (n = 28) of the patients in the non S-point group. Overweight (BMI 23 to 25) was noted in 30.0% (n = 12) and 19.2% (n = 19) of the patients and obesity (BMI over 25) was noted in 35.0% (n = 14) and 50.5% (n = 50) of the patients in the S-point group and the non S-point group, respectively. The difference in BMI distribution was statistically significant (*p* = 0.010). Table 5 shows that the mean BMI level was also different between the groups (23.41 ± 3.71 vs. 24.93 ± 3.97 in the S-point group and the non-S-point group, respectively).

**Table 4:** BMI score and anemia grade of S-point group and Non S-point group.

	**S-point bleeding (n** **=** **40)**	**Non S-point bleeding (n** **=** **99)**	***p*****-value**
**BMI**			0.010^a^
Underweight (BMI < 18.5)	6 (15.0%)	2 (2.0%)	
Normal (BMI 18.5 to 23)	8 (20.0%)	28 (28.3%)	
Overweight (BMI 23 to 25)	12 (30.0%)	19 (19.2%)	
Obesity (BMI >25)	14 (35.0%)	50 (50.5%)	
**Anemia**			0.001^a^
Normal Hb	13 (32.5%)	63 (63.6%)	
Anemia	27 (67.5%)	36 (36.4%)	
**Anemia grade**			<0.001^a^
Normal Hb	13 (32.5%)	63 (63.6%)	
Mild anemia	24 (60.0%)	24 (24.2%)	
Moderate anemia	3 (7.5%)	7 (7.1%)	
Severe anemia	0 (0%)	5 (5.1%)	

BMI, Body Mass Index; Hb, Hemoglobin.

Normal Hb, Hb 12.0–16.0 g/dL for female and 14.0–18.0 g/dL for male; Mild anemia, Hb 10 g/dL to levels within normal limits; Moderate anemia, Hb 8–10 g/dL; Severe anemia, Hb 6.5–7.9 g/dL.

a Indicate statistically significant (*p*-value < 0.05).

**Table 5:** Mean initial hemoglobin level and BMI score of S-point group and Non S-point group.

	**S-point bleeding (n** **=** **40)**	**Non S-point bleeding (n** **=** **99)**	***p*****-value**
**Initial Hb level**	12.69 (±1.99)	13.16 (±2.37)	0.266
**BMI**	23.41 (±3.71)	24.93 (±3.97)	0.039^a^

Hb, Hemoglobin; BMI, Body Mass Index.

a Indicate statistically significant (*p*-value < 0.05).

### Relationship between S-point bleeding and initial hemoglobin level

Anemia was noted in 67.5% (n = 27) of the patients in the S-point group and in 36.4% (n = 36) of the patients in the non-S-point group (Table 4). The difference was statistically significant (*p* = 0.001). Specifically, the incidence of mild anemia (60.0% vs. 24.2% in the S-point group and the non S-point group, respectively), moderate anemia (7.5% vs. 7.1%), and severe anemia (0% vs. 5.1%) showed a statistically significant difference (*p* < 0.001). Incidence of anemia, especially that of mild anemia, was higher in the S-point group than that in the non S-point group. The mean initial hemoglobin level was 12.69 ± 1.99 in the S-point group and 13.16 ± 2.37 in the non S-point group. However, this difference was not statistically significant (*p* = 0.266) (Table 5).

## Discussion

Epistaxis is one of the most common reasons for visiting the otorhinolaryngology department and needs emergent treatment.^1^ Currently, endoscopic examination has made it possible to evaluate the nasal cavity more precisely. Identifying the precise bleeding focus is important while treating epistaxis, especially in severe cases, as these cases need urgent care. The precise location of bleeding in cases of severe epistaxis is controversial. However, previous studies have pointed out that the nasal septum was a common bleeding focus in severe epistaxis.^4^, ^5^ Currently, the S-point is gaining importance as a bleeding focus in severe epistaxis. Due to its arterial nature, S-point bleeding may be mistakenly identified as posterior epistaxis such as that originating from SPA.^2^ Therefore, identifying the characteristics of patients with severe epistaxis, classified according to the bleeding focus, can be beneficial in predicting the precise bleeding focus.

In this study, we investigated the prevalence rate of S-point bleeding and the associated patient characteristics such as comorbidities, obesity, anemia, hypertension, diabetes, and dyslipidemia and compared them with the characteristics associated with non S-point bleeding. Previous study demonstrated that 6% of patients who affected by epistaxis require professional medical attention,^6^ and 5% of all patients who admitted to the hospital or treated in the emergency department due to epistaxis had posterior nasal bleeding focus.^7^ In a previous study, age distribution of patients who were admitted to hospital for epistaxis was skew deviated to left.^8^ In this study, the age distribution was not deviated though the difference was not statistically significant.

We were able to evaluate the prevalence of S-point bleeding in cases of suspected severe posterior bleeding (28.8% of non-anterior bleeding cases). The results suggest that the S-point is a frequent site of severe epistaxis. This study revealed that comorbidities such as hypertension, diabetes, and dyslipidemia did not contribute significantly to S-point bleeding. Previous studies have stated that these factors are related to epistaxis.^9^ However, those studies were different from our study because they studied the entire type of epistaxis. Kosugi et al.^2^ have identified the characteristics of patients with S-point bleeding, but could not find any differences when compared with non S-point bleeding. Recently, Loures et al.^10^ reported that the S-point was the most common bleeding site of severe epistaxis and identified that the prevalence was 28.3% as a source of severe epistaxis. Though our study was designed as retrospective, the prevalence rate was similar to that of their study.

This study has found that the BMI of S- bleeding point group showed a significant difference when compared with BMI of non-S-point bleeding group. The mean BMI of the S-point group was lower than that of the non-S-point group. Underweight patients tended to show a greater association with S-point bleeding than with non S-point bleeding. Therefore, when physicians examine severe epistaxis patients clinically, it might be helpful to examine the S-point carefully in underweight patients. In contrast, if the patient is obese, it might be helpful to check for a posterior bleeding focus. A previous study revealed that epistaxis was positively associated with obesity.^11^ However that study was different from our study because it included patients with all types of epistaxis as well as posterior nasal bleeding. Furthermore, in the past there has been no study about the relationship between BMI and epistaxis.

The incidence of anemia, especially that of mild anemia, was higher in the S-point group than in the non S-point group. In this study, a higher proportion of patients had anemia compared with previous study that revealed that 18.5% patients with posterior epistaxis had a hemoglobin level less than 12 g/dL.^12^ We suggest that mild anemia is likely to be a result of residual bleeding that was not treated completely during initial care. This finding might indicate that the primary care before visiting our hospital was not adequate, as the S-point is located in the upper septum and is difficult to evaluate primarily. If the origin of bleeding is identified, bleeding can be controlled by electric cauterization. In this study, cauterization was the most common treatment in both the groups. Therefore, routine endoscopic examination of the S-point in patients with severe epistaxis may assist in achieving good treatment results.

Nasal endoscopy helps visualize the proper bleeding site and treat epistaxis originating from an area that is not easily accessible.^13^ Development of nasal endoscopic techniques has led to accurate localization and cautery of the bleeding point, and several authors have described this technique to successfully treat the cases of epistaxis.^14^, ^15^ In this study, electric cauterization was the most common final treatment for patients in both the groups. This underlines the significance of locating the accurate bleeding focus to treat epistaxis, as locating the precise bleeding focus must precede proper electric cauterization. This study has some limitations. It was a retrospective study and we depended on the descriptions in the chart to locate the bleeding focus. In the past, the S-point was not considered a frequent bleeding focus and was not routinely checked. Hence, it is possible that the accurate bleeding focus was missed in some patients. Moreover, history taking regarding underlying diseases may have been insufficient. In addition, if the patient underwent nasal packing as an initial approach in primary and secondary facilities, it can hinder the precise identification of the bleeding site and therefore limit the classification into two groups.

Despite these limitations, we believe that our study may contribute in identifying the correct bleeding focus in severe epistaxis. Although previous studies have identified the specific site of superior septal bleeding and suspected that it may be a frequent site of epistaxis, our study determined the prevalence rates and the risks for S-point bleeding.

## Conclusions

We found that the prevalence of S-point bleeding was 28.8% of non-anterior bleeding cases in our study, and it seems to be significant. Patients with S-point bleeding had lower BMI scores than those with non-S-point bleeding, and underweight patients had a higher risk of S-point bleeding. S-point bleeding showed a significant association with anemia, which might be due to inadequate primary treatment. We expect that careful examination of the S-point in patients with severe epistaxis, especially in underweight patients, may increase treatment success.

## Data availability statement

The data that support the findings of this study are available from the corresponding author upon reasonable request.

## Ethical considerations

This study was approved by Konkuk University Hospital Institutional Review Board (Approval n° 2019-08-012-003) and Chung-Ang Universtiy Hospital Institutional Review Board (Approval n° 1912-023-19296).

## Funding

This research was supported by the Basic Science Research Program through the National Research Foundation of Korea (NRF) funded by the Ministry of Education (NRF-2016R1D1A1B01- 012705, NRF-2016R1A5A2012284).

## Conflicts of interest

The authors declare no conflicts of interest.
